# Supplementary material for: Estimating and predicting the temporal information of apartment burglaries that possess imprecise time stamps: A comparative study using eight different temporal approximation methods in Vienna, Austria
Source: PLoS One. 2021 Aug 19;16(8):e0253591. doi: 10.1371/journal.pone.0253591 (PMC8376027; doi:10.1371/journal.pone.0253591)
Supplement: Attachment — Submitted filename: Response to Reviewers.docx [file pone.0253591.s001.docx]

Reviewers' comments:

Reviewer's Responses to Questions

**Comments to the Author**

1. Is the manuscript technically sound, and do the data support the conclusions?

The manuscript must describe a technically sound piece of scientific research with data that supports the conclusions. Experiments must have been conducted rigorously, with appropriate controls, replication, and sample sizes. The conclusions must be drawn appropriately based on the data presented.

Reviewer #1: Partly

Reviewer #2: Yes

2. Has the statistical analysis been performed appropriately and rigorously?

Reviewer #1: Yes

Reviewer #2: Yes

3. Have the authors made all data underlying the findings in their manuscript fully available?

The [PLOS Data policy](https://nam04.safelinks.protection.outlook.com/?url=http%3A%2F%2Fwww.plosone.org%2Fstatic%2Fpolicies.action%23sharing&data=04%7C01%7Cmleitne%40lsu.edu%7C0808ef8eae2148a492a708d8a72841d6%7C2d4dad3f50ae47d983a09ae2b1f466f8%7C0%7C0%7C637443138657910459%7CUnknown%7CTWFpbGZsb3d8eyJWIjoiMC4wLjAwMDAiLCJQIjoiV2luMzIiLCJBTiI6Ik1haWwiLCJXVCI6Mn0%3D%7C1000&sdata=v8snp4LDnsyCFljjCpkMNfj1CHfCn7eYf27zfwJhF0s%3D&reserved=0) requires authors to make all data underlying the findings described in their manuscript fully available without restriction, with rare exception (please refer to the Data Availability Statement in the manuscript PDF file). The data should be provided as part of the manuscript or its supporting information, or deposited to a public repository. For example, in addition to summary statistics, the data points behind means, medians and variance measures should be available. If there are restrictions on publicly sharing data—e.g. participant privacy or use of data from a third party—those must be specified.

Reviewer #1: Yes

Reviewer #2: Yes

4. Is the manuscript presented in an intelligible fashion and written in standard English?

PLOS ONE does not copyedit accepted manuscripts, so the language in submitted articles must be clear, correct, and unambiguous. Any typographical or grammatical errors should be corrected at revision, so please note any specific errors here.

Reviewer #1: Yes

Reviewer #2: Yes

5. Review Comments to the Author

Please use the space provided to explain your answers to the questions above. You may also include additional comments for the author, including concerns about dual publication, research ethics, or publication ethics. (Please upload your review as an attachment if it exceeds 20,000 characters)

Reviewer #1: The manuscript “Estimating and predicting the temporal information of apartment burglaries that possess imprecise timestamps: A comparative study using eight different temporal approximation methods in Vienna, Austria” introduced two novel temporal approximation methods and evaluated apartment burglaries in Vienna, Austria, for yearly and seasonal differences. The manuscript is a methodological study with an element of a case study. The analysis the paper promises is good, but the analysis delivered is less exciting. The methods are remarkably similar to existing studies. Therefore, I suggest emphasizing and in-depth analysis of seasonal differences.

1. Both the RWA, RWAext novel methods are similar to the methods provided in another published article (doi:10.3390/ijgi9060386) . Assumptions are also similar. So the methods are not very novelty. The difference between the novel methods in this paper and the existing methods should be identified.

The retrospectively weighted aoristic (RWA) temporal approximation method proposed in this manuscript is indeed novel and has not been mentioned in “doi:10.3390/ijgi9060386”. The retrospectively weighted aoristic_ext_ (RWA_ext_) method is relatively new and is the same as the RTA_ext_ method in “doi:10.3390/ijgi9060386”.

We have clarified the difference between the novel methods in this manuscript and the existing novel methods in “doi:10.3390/ijgi9060386” throughout the revised manuscript, including the Abstract, 1. Introduction, 3.1. Research Objectives, 3.3.1. Temporal Approximation Methods, 4.4. Limitations, and 5. Conclusion.

2. Line 42: Please also include other keywords to mention the temporal approximation methods you introduced.

*“Retrospectively weighted aoristic”* and*“retrospectively weighted aoristic_ext_”* methods are added to keywords

3. Line 269: As the Euqation (1) you mentioned shows the principle of the RWA method, please also use an Equation to show the principle of the RWAext method.

The equation of the RWA_ext_ method is added to Subsection 3.3.1. Temporal Approximation Methods as Equation (2) together with a fictive example.

4. Line 278: It seems that the number of Section “3.3.1 Statistical Evaluation” was wrong.

Corrected

5. Line 336: It will be better to additionally compare approximated distributions of apartment burglaries with unknown times from 2013 to 2015 to apartment burglaries with known offense times from 2013 to 2015. I leave the final decision to the authors.

This research has two goals, first to approximate offense times of retrospective (historic) crimes that possess imprecise temporal information and second to evaluate approximated offense times of future crimes with imprecise temporal information. While the suggestion by this reviewer can be used to evaluate approximation methods of offense times for retrospective (historic) crimes with imprecise timestamps, it would not be able to evaluate approximated offense times of future crimes with imprecise temporal information. Hence this approach was not applied in this research.

6. Line 450: Authors should add other similar work to improve the scientific strength of the work. There are no references published in the past two years in the references section.

Three articles published in 2020 were added.

7. Line 519: Incomplete legend has been provided in Figure 1. And the meaning of vertical axis is not clear.

Complete legend has been provided and vertical axis has been labeled.

Start here: Reviewer #2: This study addresses the question of how to handle temporal uncertainty in the time coding of crime incidents. It compares 8 different strategies for addressing such uncertainty, and compares the results. It uses data from Vienna for these comparisons.

I like the general idea of the research question, as it is certainly an important one. It seems like the data are useful for addressing the question, although there were a few details I was unclear on.

It is useful to compare strategies to the two aoristic strategies that are included, given that they are often used in studies. I do think these are quite naïve strategies, particularly the initial aortic one that does not account for partial hours, so it might be useful to at least point out a few of the potential limitations we would expect from these strategies based on their assumptions.

A paragraph discussing the main limitation of both aoristic methods has been added to Section 1. Theoretical Background.

I found myself somewhat confused about what exactly the data provides. Is there different time information for crime incidents in different years? At points, it seemed like there was, and that fed into the strategy used. But at other points it seemed like there was not. It would be good to add a paragraph that makes it explicit what temporal information is available in which years. And which years have the “gold standard” of correct time, and how exactly is that “gold standard” determined? This should be presented in a very clear fashion, as currently the information about this seems to be spread at various points in the data section, and it was not very clear at points. This may mostly be a re-ordering of information in the data section.

More text was added to the third paragraph under Subsection 3.2. Data Preparation to make it very clear which three datasets were collected and applied in this research. Also, the two terms “precisely known time stamps” and “imprecisely known time stamps” are now used throughout the manuscript (read our answer to the following question next).

It was not clear to me at points whether the author(s) were referring to incidents with exact time of incident, incidents with a range of time period, or incidents with no time information (I’m not sure how the latter would occur; it seems like this should just have a very wide time range). But making this clearer would help the readability at points.

Two types of time stamps are used for incidents in this research. First, incidents with precisely (accurately) known time stamps (to the minute), and second, incidents with imprecisely (inaccurately) known time stamps not exceeding a 24-hour time period. Incidents with no time information are not included. The term “precisely known offense time” (or similar) is now used for the first type of time stamps and “imprecisely known offense time” (or similar) for the second type of time stamps throughout this manuscript.

I wondered if another strategy would be to use time from the beginning of the unknown time period? If a house was being cased, it might be that it would be burglarized shortly after the people left, regardless of what time that is. Just a thought.

This idea has been implemented in this study with one of the four naïve methods (*t*_start_) and interestingly performed the poorest of all eight temporal approximation methods (compare results in Tables 2 and 3).

On page 15, it was not clear to me whether the correlation reported is based on specific cases, or the distribution? I believe it’s the latter, but that should be clarified.

Correlation statistics are based on the distribution.

These are some interesting findings. It would be useful to talk about the potential generalizability of them. There are many possible issues. How generalizable are the temporal patterns of crime in this city? How generalizable is this particular crime type to others? It would be good to explicitly talk about these issues in the discussion section, as they would be important to potential users of the technique, especially if they did not have the type of data used here to calibrate the model. They would need to assume some of the patterns in the crime data here, so how reasonable would that be?

A new paragraph (now paragraph 3) is added to Section 5. Conclusion to discuss these issues.

6. PLOS authors have the option to publish the peer review history of their article ([what does this mean?](https://nam04.safelinks.protection.outlook.com/?url=https%3A%2F%2Fjournals.plos.org%2Fplosone%2Fs%2Feditorial-and-peer-review-process%23loc-peer-review-history&data=04%7C01%7Cmleitne%40lsu.edu%7C0808ef8eae2148a492a708d8a72841d6%7C2d4dad3f50ae47d983a09ae2b1f466f8%7C0%7C0%7C637443138657910459%7CUnknown%7CTWFpbGZsb3d8eyJWIjoiMC4wLjAwMDAiLCJQIjoiV2luMzIiLCJBTiI6Ik1haWwiLCJXVCI6Mn0%3D%7C1000&sdata=CqgaMPsvHTZ%2BXfbbNWZ26B%2FtGOohij8Mvqd6DtPYR3U%3D&reserved=0)). If published, this will include your full peer review and any attached files.


If you choose “no”, your identity will remain anonymous but your review may still be made public.


**Do you want your identity to be public for this peer review?** For information about this choice, including consent withdrawal, please see our [Privacy Policy](https://nam04.safelinks.protection.outlook.com/?url=https%3A%2F%2Fwww.plos.org%2Fprivacy-policy&data=04%7C01%7Cmleitne%40lsu.edu%7C0808ef8eae2148a492a708d8a72841d6%7C2d4dad3f50ae47d983a09ae2b1f466f8%7C0%7C0%7C637443138657920453%7CUnknown%7CTWFpbGZsb3d8eyJWIjoiMC4wLjAwMDAiLCJQIjoiV2luMzIiLCJBTiI6Ik1haWwiLCJXVCI6Mn0%3D%7C1000&sdata=JavAQ2i9ieSopVtrLUX1kbFszC%2FUd%2BzryJ2dEL%2Fy81k%3D&reserved=0).

Reviewer #1: No

Reviewer #2: No

[NOTE: If reviewer comments were submitted as an attachment file, they will be attached to this email and accessible via the submission site. Please log into your account, locate the manuscript record, and check for the action link "View Attachments". If this link does not appear, there are no attachment files.]

While revising your submission, please upload your figure files to the Preflight Analysis and Conversion Engine (PACE) digital diagnostic tool, [https://pacev2.apexcovantage.com/](https://nam04.safelinks.protection.outlook.com/?url=https%3A%2F%2Fpacev2.apexcovantage.com%2F&data=04%7C01%7Cmleitne%40lsu.edu%7C0808ef8eae2148a492a708d8a72841d6%7C2d4dad3f50ae47d983a09ae2b1f466f8%7C0%7C0%7C637443138657920453%7CUnknown%7CTWFpbGZsb3d8eyJWIjoiMC4wLjAwMDAiLCJQIjoiV2luMzIiLCJBTiI6Ik1haWwiLCJXVCI6Mn0%3D%7C1000&sdata=%2BrM32B2FgMoZtn5A6aooiHsrF3iHrmaeN8uw%2FFvnUtk%3D&reserved=0). PACE helps ensure that figures meet PLOS requirements. To use PACE, you must first register as a user. Registration is free. Then, login and navigate to the UPLOAD tab, where you will find detailed instructions on how to use the tool. If you encounter any issues or have any questions when using PACE, please email PLOS at [figures@plos.org](mailto:figures@plos.org). Please note that Supporting Information files do not need this step.
